# Mutational Analyses of the Influenza A Virus Polymerase Subunit PA Reveal Distinct Functions Related and Unrelated to RNA Polymerase Activity

**DOI:** 10.1371/journal.pone.0029485

**Published:** 2012-01-06

**Authors:** Yuhong Liang, Shamika Danzy, Luan Danh Dao, Tristram G. Parslow, Yuying Liang

**Affiliations:** Department of Pathology and Laboratory Medicine, Emory University, Atlanta, Georgia, United States of America; University of Cambridge, United Kingdom

## Abstract

Influenza A viral polymerase is a heterotrimeric complex that consists of PA, PB1, and PB2 subunits. We previously reported that a di-codon substitution mutation (G507A-R508A), denoted J10, in the C-terminal half of PA had no apparent effect on viral RNA synthesis but prevented infectious virus production, indicating that PA may have a novel role independent of its polymerase activity. To further examine the roles of PA in the viral life cycle, we have now generated and characterized additional mutations in regions flanking the J10 site from residues 497 to 518. All tested di-codon mutations completely abolished or significantly reduced viral infectivity, but they did so through disparate mechanisms. Several showed effects resembling those of J10, in that the mutant polymerase supported normal levels of viral RNA synthesis but nonetheless failed to generate infectious viral particles. Others eliminated polymerase activity, in most cases by perturbing the normal nuclear localization of PA protein in cells. We also engineered single-codon mutations that were predicted to pack near the J10 site in the crystal structure of PA, and found that altering residues K378 or D478 each produced a J10-like phenotype. In further studies of J10 itself, we found that this mutation does not affect the formation and release of virion-like particles per se, but instead impairs the ability of those particles to incorporate each of the eight essential RNA segments (vRNAs) that make up the viral genome. Taken together, our analysis identifies mutations in the C-terminal region of PA that differentially affect at least three distinct activities: protein nuclear localization, viral RNA synthesis, and a *trans*-acting function that is required for efficient packaging of all eight vRNAs.

## Introduction

The RNA-dependent RNA polymerase of influenza A virus is a heterotrimeric protein complex comprised of three virally-encoded subunits, designated PA, PB1, and PB2 [1], [2]. Localized within the nuclei of infected host cells, this polymerase trimer is essential for synthesizing all influenza RNA species. Its functions include replication of the eight different uncapped, non-polyadenylated, negative-sense RNA segments (vRNAs) that make up the viral genome, and of the positive-sense replicative intermediates known as cRNAs. It also transcribes all viral mRNAs, using a biochemically distinct process that is initiated through polymerase-mediated cap-snatching from host mRNAs and yields capped, polyadenylated viral transcripts [1]. The polymerase is also a key structural constituent of influenza virion particles, within which a single polymerase trimer is believed to be associated stably with each of the packaged vRNAs, binding simultaneously to its paired 3′ and 5′ ends [3], [4]. This stable binding of one polymerase trimer onto each vRNA occurs within the host-cell nucleus and, together with the binding of viral nucleoprotein (NP) and other factors, gives rise to a ribonucleoprotein complex [5], which serves as the substrate for nucleocytoplasmic export, intracellular trafficking, and packaging of the vRNAs into nascent virions.

Biochemical and genetic studies of the polymerase subunits have helped delineate the roles that each of these proteins plays in viral RNA synthesis. PB2 has a cap-binding motif that binds to the 5′ caps of host-cell mRNAs [6], [7]. PB1 has RNA-dependent RNA polymerase activity and binds to the conserved terminal sequences of the vRNA and cRNA [8], [9], [10]. PA has been shown to interact directly with the PB1 subunit of the intact trimer complex, and plays an essential role in viral RNA synthesis [11], [12], [13], [14], [15], [16], [17], [18], [19]. PA consists of two domains connected by a linker [20]. Although no full-length structure of PA has yet been reported, the high-resolution structures of its two halves in isolation provide numerous insights, including atomic-level details of the binding interactions between its C-terminal domain and the PB1 subunit in the trimeric polymerase complex, as well as the unexpected discovery that the cap-endonuclease enzymatic site resides within its N-terminal domain [21], [22], [23], [24]. These structural findings offer a new context in which to revisit data from earlier mutational studies that probed the functional topology of PA.

Our previous mutational analysis of PA identified a unique substitution mutant designated J10, involving residues G507 and R508 in the C-terminal half of the protein, whose phenotype unexpectedly suggested that PA has a key role in viral replication unrelated to polymerase activity [19]. In particular, the J10 mutant appeared to have little or no effect on synthesis of any of the three classes of viral RNA, yet it completely abolished the production of infectious virions [19]. In this study, we have carried out further mutational analysis of regions that lie near J10 in the known crystallographic structure of C-terminal PA. Our studies yielded groups of mutants with distinct phenotypic defects. One set of PA mutants proved defective in nuclear localization, which presumably accounts for their lack of RNA polymerase activity. Other mutants lacked polymerase enzymatic function despite being properly localized. Still others exhibited a J10-like phenotype, in that they abolished viral infectivity without any apparent effect on viral RNA synthesis. On further analysis of J10 itself, we found that this mutation does not affect the production or release of virus-like particles but instead decreases viral RNA packaging into these particles. Our studies thus reveal that mutations within the C-terminal domain of PA can differentially affect PA localization, viral polymerase activity, and a third, novel function related to viral RNA packaging.

## Materials and Methods

### Cells

Cell lines were obtained from American Type Culture Collection (ATCC). 293T (human epithelial kidney cells) cells were grown in Dulbecco's modified Eagle's medium (DMEM with high glucose) supplemented with 10% heat-inactivated fetal bovine serum (FBS). Madin-Darby canine kidney (MDCK) and Madin-Darby bovine kidney (MDBK) cells were maintained in Eagle's minimal essential medium (MEM) supplemented with 5% FBS. After infection with either influenza virus or viral-like particles (VLPs), MDCK and MDBK cells were grown in L-15 medium containing 15 mM HEPES, pH7.5, nonessential amino acids, 0.75 g of NaHCO_3_ per liter, and 0.125% (w/v) of bovine serum albumin.

### Plasmids

The 17-plasmid [25] influenza A virus reverse genetic systems were obtained from Y. Kawaoka (University of Wisconsin). The luciferase reporter construct cNA-LUC was previously described [19]. PA 66-G-50, PB1 66-G-50, and PB2 0-G-100 are GFP reporter constructs carrying packaging signals from the PA, PB1, and PB2 vRNAs, respectively, and have been described elsewhere [26]. Mutations of the PA gene were created by PCR-based mutagenesis on pHH21-PA using primers containing the intended changes. The protein-expression vectors encoding each PA mutant were generated by PCR amplification of the PA open-reading frame from the corresponding pHH21-PA constructs and cloned into the pcDNA3.1 vector. Primer sequences will be provided upon request. All mutations were confirmed by DNA sequencing.

### Generation of influenza A viruses harboring PA mutations

293T cells in 6-well plates were transfected with 17 plasmids together to generate infectious influenza A/WSN viruses [25], including either the wild-type (WT) pHH21-PA vector one of its mutant counterparts, using TransIT-LT1 transfection reagent (Panvera, Madison, Wisconsin) according to the manufacturer's protocol and as described before [19]. Cell media were replaced at 6 h post-transfection with Opti-MEM containing 0.3% BSA and 0.01% FBS. Supernatants were collected at 48 h post-transfection for virus titer determination by plaque assay.

### Evaluation of viral RNA transcription in a 5-plasmid transfection system

293T cells were transfected with 0.1 µg of the LUC reporter construct cNA-LUC [19], together with 0.25 µg of each of the protein-expression vectors encoding PB2, PB1, and NP, and with 0.25 µg of the protein-expression vector encoding either WT or mutant PA protein. A β-galactosidase (β-gal) expression plasmid (Invitrogen) was included as an internal control for normalizing transfection efficiency. At 24 h post-transfection, cells were harvested for LUC assay and normalized to β-gal activity. LUC activity and β-gal activity were measured using firefly LUC assay system (Promega) and β-gal enzyme assay system (Promega), respectively.

### Evaluation of viral RNA synthesis by primer-extension assay

The 5-plasmid assay was conducted as described above. Total RNA was harvested from the transfected cells and analyzed by the established primer extension assay for the level of three viral RNA species, cRNA, vRNA, and mRNA. In brief, strand-specific primers were 5′-end-labeled with [gamma-^32^P]ATP and annealed with total RNAs for reverse transcription. The primer extension products (with expected lengths of 75 nt for vRNA, 85 nt for cRNA, and 93–96 nt for mRNA) were precipitated and separated on a 10% urea-denaturing polyacrylamide gel. The gel was dried and autoradiographed.

### Generation of replication-defective viral-like particles (VLPs)

293T cells were transfected with the 17 plasmids required to generate replication-competent virions, except that either the WT or the J10-mutant forms of both the protein- and vRNA-encoding vectors for PA were included, and with one vRNA-expression vector (PA, PB1 or PB2) replaced by a vector encoding the corresponding packaging-competent GFP construct PA-GFP (PA 66-G-50), PB1-GFP (PB1 66-G-50) or PB2-GFP (PB2 0-G-100).

For metabolic labeling, the transfected cells were pre-incubated with media containing dialyzed FBS for 30 min prior to the addition of ^35^S labeling mix (PerkinElmer). After 24 h, the supernatants were collected, filtered through 0.45 µM filters, and enriched for VLPs using either chicken erythrocytes or polyclonal anti-H1N1 antiserum (Fitzgerald Inc., MA). The corresponding cell lysates were prepared and immunoprecipitated with anti-H1N1 antiserum. Enriched VLPs from the supernatants or the viral proteins in the cell lysates were then analyzed by SDS-PAGE and detected by autoradiography.

To quantify packaging efficiency, VLPs were prepared without metabolic labeling. At 48 h post-transfection, supernatants from transfected 293T cells were collected, filtered through 0.45 µM filters, and then used either for flow cytometric analysis or for real-time RT-PCR quantification. For flow cytometric analysis, aliquots of supernatants were used to infect MDCK cells, together with helper A/WSN virus at moi of 0.1. The GFP-transferring units per ml were then determined by flow cytometric analysis of the infected MDCK cells at 18 hpi.

### Quantitation of VLP-incorporated vRNA segments by real-time RT-PCR

We incubated transfection supernatants with 1 µl of micrococcal nuclease at 37°C for 30 minutes, prior to extracting viral RNA by Qiagen viral RNA extraction kit. Total RNA was cleared of possible plasmid DNA contamination by incubating samples for 30 min at 37°C with DNase I, which was then inactivated by heating samples to 85°C for 15 min. Reverse transcription was conducted using influenza-specific primer and the SuperScript III first-strand synthesis system (Invitrogen). Quantitative real-time PCR was performed in a 20-µl reaction with gene-specific primers as described previously [27]. Primer sequences will be provided upon request.

## Results

### Mutational analysis of residues 497–518 of PA in viral infectivity

**Figure 1:**
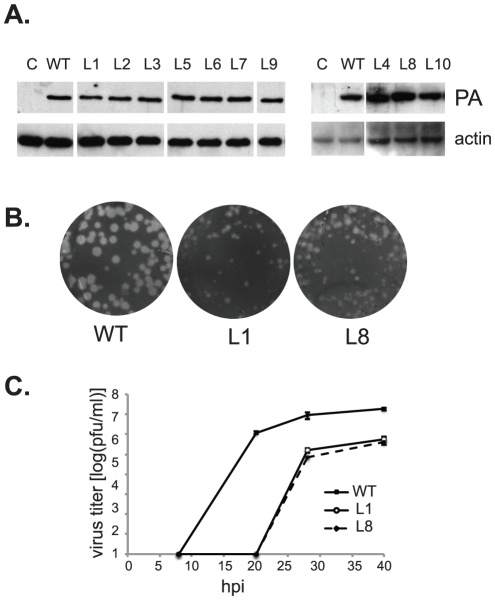
Mutational analysis of residues 497–518 of PA on viral growth. (**A**) Expression level of the PA mutants in the transfected 293T cells was analyzed by western blot analysis using anti-PA polyclonal antibody. (**B**) Plaque formation on MDCK cells by wild-type (WT), recombinant mutant L1 (K497A, T498A), and L8 (N513A, D514A). (**C**) Growth curve analysis of WT, L1, and L8 on MDCK cells at moi of 0.001.

We have reported that the bi-codon mutation J10 (G507A-R508A) in PA has little or no apparent effect on viral RNA synthesis but severely impairs infectivity and viral particle yield in a spreading-infection assay [19]. This implied a novel function for PA protein in the viral life cycle, whose exact nature was unknown. Though located within the C-terminal domain, J10 does not coincide with the PB1-interaction site or other functional residues mapped previously in this domain [13], [14], [21], [22], [28], [29]. We therefore created additional scanning mutations to interrogate the regions flanking the J10 site. As in our earlier study, each was a di-codon substitution mutation designed to replace a pair of consecutive residues with alanines; a total of 10 mutants (designated L1–L10) were created that together scanned residues 497–518 of PA (**Table 1**). Western blot analysis confirmed that, when plasmids encoding these mutants were transiently expressed in 293T cells, each of the eight mutants yielded protein of comparable size and abundance to wild type PA (**Figure 1A**). When recombinant viruses carrying these mutations were assembled in the 17-plasmid A/WSN system, however, only L1 and L8 were able to support plaque formation, with a greatly reduced virus titer observed for L8 (**Table 1**). All other mutations yielded no detectable plaque-forming virus, apart from rare wild-type revertants. Both L1- and L8-containing viruses produced small, cloudy plaques (**Figure 1B**), and showed delayed growth kinetics as compared to wild type (**Figure 1C**), suggesting that each had a partial defect in spreading infection. Collectively, these results reveal that mutations throughout a 21-amino-acid region (residues 497–518) in PA protein that includes the J10 site can impair viral infectivity.

**Table 1:** Rescue of recombinant viruses with mutations within residues 497–518 of PA.

Virus	Mutations	Plaque Titer	Plaque Morphology
**WT**		1.0E+6	Large, Clear
**L1**	K497A, T498A	3.4E+5	Small, unclear
**L2**	N499A, L500A	0	N/A
**L3**	Y501A, G502A	0	N/A
**L4**	F503A, I504A	0	N/A
**L5**	I505A, K506A	0	N/A
**L6**	S509A, H510A	0	N/A
**L7**	L511A, R512A	0	N/A
**L8**	N513A, D514A	7.2E+3	Small, unclear
**L9**	T515A, D516A	0	N/A
**L10**	V517A, V518A	0	N/A

N/A, not applicable.

### Mutational analysis of residues 497–518 of PA in viral RNA synthesis

**Figure 2:**
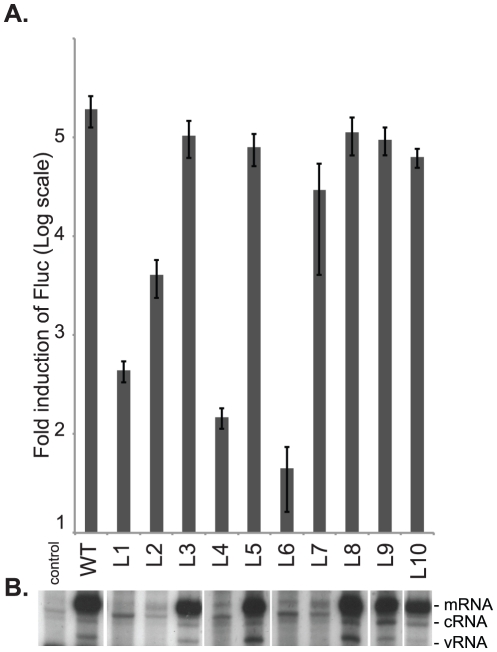
Mutational analysis of residues 497–518 of PA on viral RNA synthesis. (**A**) The effects of PA mutants on viral RNA synthesis were analyzed in a 5-plasmid system. Fold induction of luciferase activity over the control was shown in log scale. Results shown are the average of at least 3 independent experiments with error bars representing standard deviation. (**B**) The levels of all three viral RNA species were analyzed by primer extension assay.

We then used a well-established 5-plasmid transfection assay to determine whether any of the foregoing PA mutations affected RNA-synthesis activity of the viral polymerase trimer. For this assay, 293T cells are transfected transiently with plasmids that encode, respectively, a luciferase-reporter vRNA and the four influenza proteins (PA, PB1, PB2, and NP) that are minimally required to replicate and transcribe it. When used in place of wild-type PA in the 5-plasmid assay (**Figure 2A**), five mutants (L3, L5, L8, L9, and L10) were able to support luciferase expression at levels comparable to that of the wild type. By contrast, mutant L7 supported only ∼15% of wild-type luciferase expression, and mutants L1, L2, L4, and L6 showed little or none, indicating that polymerase complexes containing any of the latter five mutants are defective in RNA synthesis. That conclusion was directly confirmed by the results of a primer-extension assay that directly quantifies the levels of all three viral RNA species produced from the luciferase reporter in these same cells (**Figure 2B**). In the primer-extension assay, cells that expressed mutants L3, L5, L8, L9, and L10 each produced levels of vRNA, cRNA, and mRNA comparable to those seen with wild-type PA, whereas mutants L1, L2, L4, L6, and L7 were defective in production of all three RNA species. Taken together, these results demonstrate that whereas some of the mutations within this region of PA that disable infectivity are associated with impaired viral RNA synthesis, others, like J10, are not.

### Nuclear localization of PA 497–518 mutants

**Figure 3:**
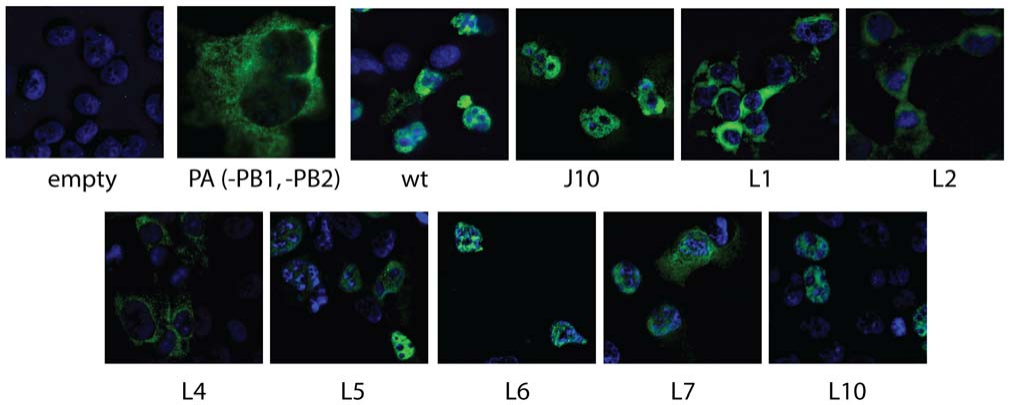
Nuclear localization of PA constructs. COS1 cells were transfected with expression vector of wild-type or mutant PA proteins, with or without (-PB1, -PB2) expression vectors of PB1 and PB2. Immunofluorescence analysis was conducted to detect the PA protein by monoclonal antibody 3G5 (kindly provided by Dr. Palese, Mount Sinai), with nucleus stained with DAPI.

Because all synthesis of influenza viral RNAs takes place within the nucleus, we asked whether any of our five PA mutations that abolished RNA polymerase activity did so by interfering with the normal nuclear localization of the polymerase trimer complex. To that end, we used a PA-specific monoclonal antibody (3G5, generously provided by Dr. Peter Palese) to observe the subcellular localization of PA through indirect immunofluorescence. In 293T cells that had been transiently transfected with a PA-protein-expression vector alone, we found that PA was exclusively cytoplasmic (**Figure 3**). When co-expressed along with PB1 and PB2, however, PA was found exclusively in the nucleus, in keeping with earlier evidence that nuclear retention of PA depends mainly on its physical interaction with other polymerase subunits, particularly PB1 [30], [31], [32], [33]. We found that, like wild-type PA, the J10, L6, and L10 mutants also localized within the nucleus when co-expressed with PB1 and PB2, but that the L1, L2, and L4 mutants remained in the cytoplasm, and that L7 was distributed in both compartments (**Figure 3**). This indicates that the defective activity of polymerases containing the L1, L2, L4, or possibly L7 mutants of PA is associated with defective nuclear localization, whereas the polymerase defect produced by mutation L6 occurs independently of any evident localization defect. Of note, L10 and J10, representing the class of mutants that severely impair infectivity without affecting RNA synthesis, both exhibited the wild-type pattern of nuclear localization

### Mutational analysis of PA residues 361–480

**Figure 4:**
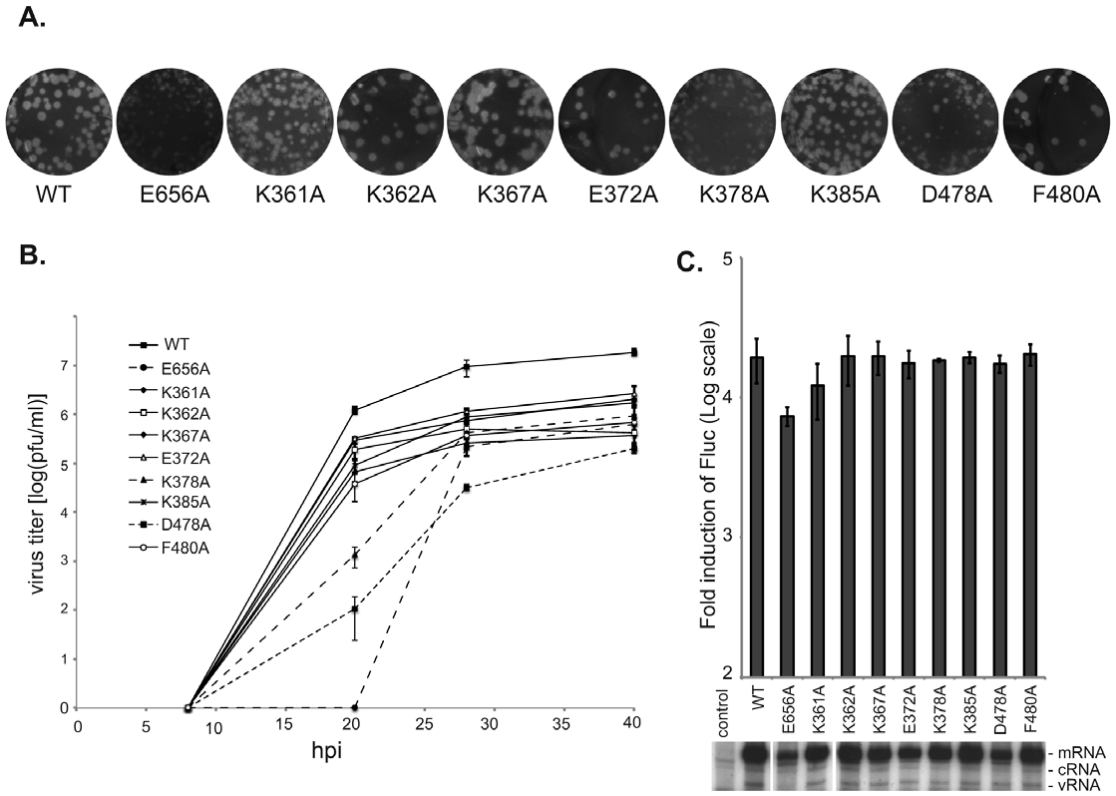
Characterization of additional single alanine substitution at charged or bulky residues in the C-terminal region. (**A**) Plaque formation on MDCK cells. Effects of PA mutations on the polymerase activity. (**B**) Growth curve analysis of recombinant viruses on MDCK cells at moi of 0.001. (**C**) The effects of PA mutants on viral RNA synthesis were analyzed in a 5-plasmid system. Fold induction of luciferase activity over the control was shown in log scale. Results shown are the average of at least 3 independent experiments with error bars representing standard deviation. The levels of all three viral RNA species were analyzed by primer extension assay.

Guided by the published crystallographic structure of the PA C-terminal domain [21], [22], we then engineered additional point mutants targeting residues that are relatively distant from J10 in the primary sequence but pack close to it in the folded native state. We targeted eight individual residues within positions 361–480, in each case substituting alanine for a single charged, polar, or bulky native residue (i.e., K361, K362, K367, E372, K378, K385, D478, and F480); in addition, we recreated the mutant E656A which had been reported by other investigators to form tiny plaques despite normal viral RNA synthesis [13], a phenotype reminiscent of J10. Each of these mutants was then used in place of wild-type PA in the 17-plasmid virus-assembly system (**Table 2**). Consistent with the earlier report, E656A yielded a virus titer more than 2 logs lower than wild type, and it formed tiny plaques (**Figure 4A**). K378A gave indistinct plaques and a 25-fold reduced titer, and D478A produced a mixture of large and small plaques. None of the other tested mutants had a substantial effect on plaque titer or morphology (**Table 2** **and** **Figure 4A**).

**Table 2:** Rescue of recombinant viruses with single alanine substitution in the C-terminal region of PA.

Virus	Mutations	Plaque Titer	Plaque Morphology
**WT**		1.0E+6	Large, Clear
**L11**	E656A	9.40E+03	Small, unclear
**L12**	K361A	2.80E+06	Large
**L13**	K362A	7.60E+05	Large
**L14**	K367A	6.00E+05	Large
**L15**	E372A	1.32E+06	Large
**L16**	K378A	3.80E+04	Medium, unclear
**L17**	K385A	2.20E+06	Large
**L18**	D478A	2.60E+05	Mixture
**L19**	F480A	2.00E+05	Large

Further characterization of these mutants was obtained through growth-curve analysis on MDCK cells (**Figure 4B**). At 20 hpi, when wild-type virus had reached a titer of 10^6^ pfu/ml, the titers of K378A and D478A were 3 and 4 fold lower, respectively, and E656A was not yet producing detectable plaques. At 28 hpi, titers of E656A and K378A each lagged the wild-type by ∼50 fold, while that of D478A lagged by 300 fold. The growth of all three mutants lagged by 20–1,000 fold at 40 hpi. The remaining mutants had only minor defects, associated with ∼10-fold decreased growth throughout the infection.

When tested for polymerase activity using the 5-plasmid assay, E656A was the only mutant in this group that showed any defect, as evidenced by modest reductions both in luciferase expression and in RNA synthesis as measured by the primer-extension assay (**Figure 4C**). In summary, among this group of nine single-codon mutations targeting residues that lie close to the J10 locus in the native PA structure, we found two (K378A and D478A) that markedly reduced infectivity without compromising RNA synthesis – the phenotype shared by L3, L5, L8, L9, and L10, and first observed in J10.

### The J10 mutation does not inhibit influenza virus-like particle assembly

Our initial characterization of J10 had shown that it imparts a profound quantitative defect in influenza particle production in a spreading-infection assay, where the yield of particles is normally amplified exponentially by cell-to-cell transmission. To determine the effect of J10 on virus production in individual cells, we therefore examined its properties in a single-round transfection assay, where transmission does not occur. This was achieved by using a modified 17-plasmid transfection assay to generate non-infectious virus-like particles (VLPs) in which one of the native vRNAs was replaced by a packaging-competent vRNA reporter that encoded GFP; these VLPs could be released into the supernatant and quantified by their ability to transduce GFP into MDCK cells, but were incapable of initiating new rounds of infection. We have previously reported three such GFP-reporter vRNAs, derived from the PA, PB1, and PB2 vRNAs, respectively, that are incorporated into virion particles with efficiencies comparable to those of the wild-type vRNAs [26]. For the present study, we assembled VLPs that incorporated each of these three reporters individually in place of the corresponding vRNA-expression vector, in the presence of either wild-type PA or the J10 mutant. The results described below were substantially identical regardless of which reporter construct was used.

**Figure 5:**
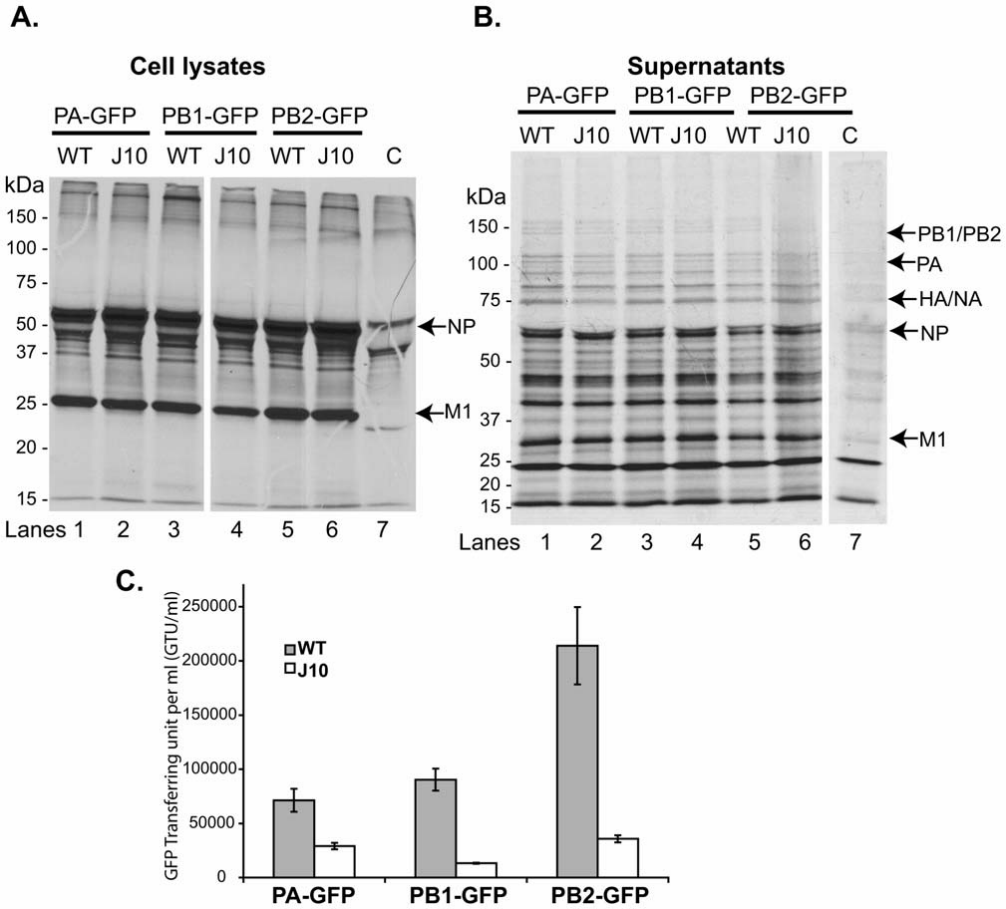
PA J10 mutant does not affect the formation of VLPs but decreases viral RNA packaging efficiency. The 293T cells were transfected with the 17-plasmid system to reconstitute the replication-defective VLPs, in which a full-length vRNA construct was replaced with a corresponding vRNA segment that encodes GFP and is packaged efficiently (PA-GFP, PB1-GFP, and PB2-GFP) [26], with either the WT or J10 mutant PA (for both the RNA and protein-encoding vectors). The cells were metabolically labeled with ^35^S for 24 h. (**A**) Cell lysates were immunoprecipitated with anti-H1N1 antiserum and separated by SDS-PAGE. The most abundant viral proteins, M1 and NP, are highlighted by arrows. C, negative control of which the PA plasmids were omitted from the transfection. (**B**) Supernatants were VLP-enriched by either chicken erythrocytes or anti-H1N1 antiserum and separated by SDS-PAGE. Only the chicken erythrocytes enriched VLPs are showing here. The viral proteins are highlighted by arrows. C, negative control of which the PA plasmids were omitted from the transfection. (**C**) Comparison of WT and J10 PA proteins in reporter vRNA packaging. The supernatants containing the replication-defective VLPs, prepared in the absence of metabolic labeling, were collected at 48 h post-transfection and used to infect fresh MDCK cells, with the helper virus at moi of 0.1. The infected MDCK cells were analyzed by flow cytometry for GFP expression at 18 hpi. The GFP-transferring unit per ml is shown.

To assay VLP production and composition, we incubated producer 293T cells continuously with ^35^S-MetCys after transfection with the 17-plasmid system, then harvested both cell lysates and supernatants at 24 h, enriched VLPs from the supernatants using density-sedimentation followed either by immunobead precipitation with polyclonal anti-H1N1 antiserum or by hemagglutination on chicken erythrocytes, and then analyzed the constituent proteins by SDS-PAGE and autoradiography. As shown in **Figure 5A**, biosynthetically radiolabelled NP, M1, and other proteins were readily detectable in producer-cell lysates that had been immunoprecipitated by anti-H1N1 antiserum; as expected, their concentrations remained similar regardless of whether a PA, PB1, or PB2 vRNA reporter had been transfected, and regardless of whether the wild-type PA protein-expression vector or its J10 mutant was used. When we then analyzed the corresponding VLP-enriched supernatants (**Figure 5B**) from these various transfectants, we again detected several radiolabelled proteins of presumably viral origin (indicated by arrows), and again found that their concentrations did not differ appreciably between J10 and wild-type PA transfectants, regardless of the vRNA reporter used. These data indicate that the J10 and wild-type PA proteins can each support the production and release of VLPs, in roughly comparable amounts and with apparently similar protein compositions.

### The J10 mutation impairs the packaging of all eight influenza vRNAs

We then asked whether the wild-type and J10-containing VLPs differed in their ability to package viral RNAs. As an initial test, we prepared VLP-containing 293T transfection supernatants as described in the preceding section, but without radiolabelling. Equivalent volumes of those supernatants were then used to infect MDCK monolayers, with the addition of excess helper virus to ensure that functional influenza polymerase activity was expressed in the target cells. Flow cytometry was then used as described [26], [27] to enumerate GFP-expressing MDCK cells, and the yield of GFP-positive cells per ml of transfection supernatant was calculated as a measure of the efficiency with which the VLPs packaged and transduced each vRNA reporter.

As depicted in **Figure 5C**, this GFP transduction assay indicated that, for each of the three vRNA reporters we tested, J10-containing VLPs packaged vRNA significantly less efficiently than did their wild-type counterparts. The extent of the apparent packaging defect (expressed as the ratio of wild-type to J10 transductants) was estimated in this assay as 2, 7, and 6 fold, respectively, for the PA, PB1, and PB2 reporters (**Figure 5C**).

Because the results of the transduction assay could potentially be influenced by events in the target cells, however, we sought to verify and extend these findings through direct analysis of VLPs. To that end, we prepared VLPs containing either wild-type or J10 PA, in the presence of the PB2 vRNA GFP reporter, enriched these particles by sucrose-gradient centrifugation, and digested them with micrococcal nuclease to remove any contaminating, unpackaged RNA. We then extracted RNA from these VLP preparations and assayed the concentrations of individual, authentic, packaged vRNAs using real-time quantitative PCR [34]. As shown in **Table 3**, we found that the GFP reporter, which served as a proxy for PB2 vRNA, was packaged only 17% as efficiently by J10-containing particles as by wild-type VLPs, corresponding to the ∼6-fold decrease in GFP transduction (**Figure 5C**). The mutant particles, moreover, also proved defective in packaging each of the seven authentic vRNAs, when compared to wild-type particles, exhibiting relative packaging efficiencies that ranged from 53% and 46% for the NS and NP segments, respectively, to as low as roughly 6–7% for the HA and M segments (**Table 3**). These data strongly suggest that the J10 mutation causes a generalized defect in the packaging efficiency of all eight influenza vRNA segments.

**Table 3:** Real time quantitative analysis of RNA segments in VLPs.

	PB2-GFP	PB1	PA	NP	HA	NA	M	NS
**WT**	100.0%	100.0%	100.0%	100.0%	100.0%	100.0%	100.0%	100.0%
**J10**	17.0%	16.0%	39.0%	46.0%	7.0%	39.0%	5.7%	53.0%

Results shown are representative data of two experiments.

## Discussion

**Figure 6:**
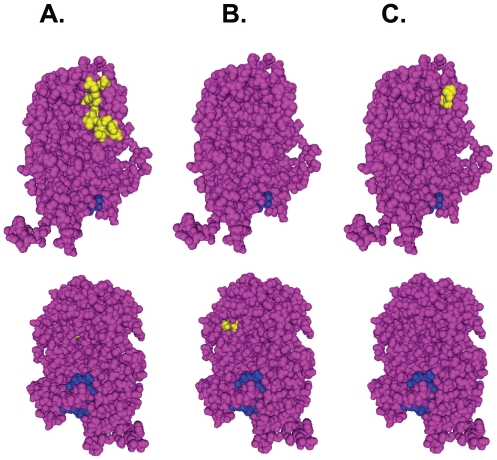
Localization of the mutants on the PA 3-D structure. The structure of the PA C-terminal region (PDB ID: 2ZNL) is shown in magenta, with the bound PB1 peptide shown in blue. (**A**) Residues corresponding to J10 and other J10-like mutants (L3, L5, L8, L9, L10, and D478) are highlighted in yellow. K378 is not shown as it is localized to an unresolved region. The position of J10 site is indicated by arrow. (**B**) Residues corresponding to L1, L2, and L4 mutations, which affect PA nuclear localization, are highlighted in yellow. (**C**) Residues of L6 mutant, which is localized to the nucleus but defective in all viral RNA synthesis, are highlighted in yellow.

Our previous discovery of a novel, lethal phenotype for mutant J10 (G507A-R508A) in the C-terminal half of PA [19] raised the possibility that PA protein plays another essential role in influenza biology that is unrelated to viral RNA synthesis. The residues altered by this J10 mutation lie in a region of the C-terminal domain that does not clearly coincide with other known functional regions, such as the endonuclease active site and PB1-interacting domain (**Figure 6A**). In the present study, we have examined a new panel of alanine substitutions designed to interrogate residues situated near the J10 site in the primary sequence or the native folded conformation of PA (mutational effects are summarized in **Table 4**). Our analyses provide several insights into structure-function relationships in this essential influenza protein.

**Table 4:** Mutational effects on viral infectivity and PA polymerase activity.

Mutants	Residues	Infectivity^1^(virus titer at 20/28/40 hpi)	RNA synthesis^2^	PA localization^3^
**WT**		++++/++++/++++	++++	Nuc
**J10**	G507A, R508A	−	++++	Nuc
**L1**	K497A, T498A	−/++/++	+	Cyt
**L2**	N499A, L500A	−	++	Cyt
**L3**	Y501A, G502A	−	++++	n/a
**L4**	F503A, I504A	−	+	Cyt
**L5**	I505A, K506A	−	++++	Nuc
**L6**	S509A, H510A	−	−	Nuc
**L7**	L511A, R512A	−	+++	Nuc
**L8**	N513A, D514A	−/+/++	++++	n/a
**L9**	T515A, D516A	−	++++	n/a
**L10**	V517A, V518A	−	++++	Nuc
**L11**	E656A	−/++/++	++++	n/a
**L12**	K361A	++/++/++	++++	n/a
**L13**	K362A	+++/++/++	++++	n/a
**L14**	K367A	+++/++/+++	++++	n/a
**L15**	E372A	+++/+++/+++	++++	n/a
**L16**	K378A	+/++/++	++++	n/a
**L17**	K385A	++/+++/+++	++++	n/a
**L18**	D478A	+/+/+	++++	n/a
**L19**	F480A	++/++/++	++++	n/a

1 Infectivity of viable recombinant virus is defined by viral titers at 20, 28, and 40 hpi time points during viral growth kinetic analyses (Fig. 1C and Fig. 4B). ++++, WT and <0.5 log difference; +++, lower than WT by 0.5–1 log; ++, lower than WT by 1–2 log; +, lower than WT by >2 log; −, no virus. If no viable virus is rescued, infectivity is defined as −.

2 The level of RNA synthesis is determined by the 5-plasmid assay (Fig. 2 and Fig. 4C). ++++, >20% WT; +++, 2–20% WT; ++, 0.1–2% WT; +, <0.1% WT; −, no detectable RNA synthesis.

3 Subcellular localization of PA protein in the presence of PB1 and PB2 proteins (Fig. 3). Nuc, nucleus; Cyt, cytoplasm; n/a, data not available.

Several mutations we tested (i.e., L1, L2, L4, L6, and L7) abolished most or all of the RNA synthetic activity of polymerase complexes that contained them, impairing the production of all three classes of influenza RNAs. Further inspection revealed that the polymerase defects of L1, L2, and L4 are associated with a failure of nuclear localization of PA, even in the presence of wild-type PB1 and PB2 proteins. Previous research has indicated that PA nuclear localization is dependent upon its interaction with PB1 [32], presumably during or after assembly of the polymerase heterotrimer. Although the residues altered by the L1, L2, and L4 mutations are not part of the PA-PB1 protein interface that was identified in a recent co-crystal structure (Figure 6B) it should be noted that this structure included only the first 15 residues of PB1 [21], [22]; hence, these three mutations might signify a second, separate contact surface for full-length PB1 or, alternatively, might influence some other aspect of polymerase complex assembly. The L6 mutant (S509A, H510A), by contrast, localizes normally but profoundly impairs viral RNA synthesis, suggesting an effect on polymerase activity *per se*. This is consistent with a previous study [35] showing that insertion of a single serine between residues S509 and H510, which are normally exposed together on the surface of PA (**Figure 6C**), yielded a dominant-negative PA mutant that suppressed viral RNA production.

Of particular note, we have also identified several other mutants that recapitulate the phenotype of J10, in that they dramatically reduce or eliminate viral infectivity without impairing the ability of the viral polymerase complex to replicate or transcribe viral RNAs. Our finding that at least eight additional, non-overlapping mutations (i.e., L3, L5, J10, L8, L9, L10, K378, and D478) all share these properties serves to validate our initial description of the phenotype, confirms that it is not unique to J10, and suggests the existence of a “J10-like” functional domain that may extend over at least 14 non-contiguous amino-acid residues in the PA protein. When mapped to the crystal structure of the PA C-terminal region (**Figure 6A**), those residues (with the exception of K378, which lies in an ill-defined portion of the structure) cluster closely together and potentially form a highly polar surface platform that might be essential for the putative novel function of PA.

Using the J10 mutation as the prototype, we have further demonstrated that this novel class of mutations does not affect the yield or protein compositions of influenza VLPs in a single-round assay, nor their release from cells (**Figure 5B**). Strikingly, however, the particles assembled in the presence of J10 were found to contain significantly reduced quantities of each of the eight genomic RNA segments, regardless of whether those segments were assayed by reporter transduction (**Figure 5C**) or by direct quantitation of individual, authentic vRNAs extracted from VLPs (**Table 3**). The finding that all eight vRNAs are affected implies a general role for PA in vRNA packaging. Indeed, both the magnitude of the effect on any given vRNA, and the extent of variation among vRNAs, are reminiscent of the packaging defects that have been observed following certain alterations of the vRNAs themselves. Importantly, although the reduction in packaging of individual segments may appear modest, their combined effects are fully sufficient, in principle, to account for the severe infectivity defect we observe in J10-containing virus: Assuming that the probability of packaging all eight vRNAs is proportional to the product of their individual packaging efficiencies, our data suggest that the relative likelihood of acquiring a complete genome is (0.17×0.16×0.39×0.46×0.07×0.39×0.06×0.53 = ) approximately 4×10^−6^ for J10-containing VLPs, or roughly six orders of magnitude lower than for wild-type influenza particles. These results therefore imply that PA is itself an essential *trans*-acting factor for packaging influenza vRNAs. Further studies will be needed to determine the mechanism by which the putative “J10-like” region of the PA protein influences vRNA packaging, as well as its potential as a target for new antiviral drugs.

## References

[1] Lamb RA, Krug RM, Fields BN, Knipe DM, Howley PM (2003). Orthomyxoviridae: the viruses and their replication.. Fields virology. 4th ed.

[2] Area E, Martin-Benito J, Gastaminza P, Torreira E, Valpuesta JM (2004). 3D structure of the influenza virus polymerase complex: localization of subunit domains.. Proc Natl Acad Sci U S A.

[3] Murti KG, Webster RG, Jones IM (1988). Localization of RNA polymerases on influenza viral ribonucleoproteins by immunogold labeling.. Virology.

[4] Klumpp K, Ruigrok RW, Baudin F (1997). Roles of the influenza virus polymerase and nucleoprotein in forming a functional RNP structure.. Embo J.

[5] Coloma R, Valpuesta JM, Arranz R, Carrascosa JL, Ortin J (2009). The structure of a biologically active influenza virus ribonucleoprotein complex.. PLoS Pathog.

[6] Blaas D, Patzelt E, Kuechler E (1982). Cap-recognizing protein of influenza virus.. Virology.

[7] Ulmanen I, Broni BA, Krug RM (1981). Role of two of the influenza virus core P proteins in recognizing cap 1 structures (m7GpppNm) on RNAs and in initiating viral RNA transcription.. Proc Natl Acad Sci U S A.

[8] Biswas SK, Nayak DP (1994). Mutational analysis of the conserved motifs of influenza A virus polymerase basic protein 1.. J Virol.

[9] Braam J, Ulmanen I, Krug RM (1983). Molecular model of a eucaryotic transcription complex: functions and movements of influenza P proteins during capped RNA-primed transcription.. Cell.

[10] Gonzalez S, Ortin J (1999). Distinct regions of influenza virus PB1 polymerase subunit recognize vRNA and cRNA templates.. Embo J.

[11] Perez DR, Donis RO (1995). A 48-amino-acid region of influenza A virus PB1 protein is sufficient for complex formation with PA.. J Virol.

[12] Gonzalez S, Zurcher T, Ortin J (1996). Identification of two separate domains in the influenza virus PB1 protein involved in the interaction with the PB2 and PA subunits: a model for the viral RNA polymerase structure.. Nucleic Acids Res.

[13] Fodor E, Crow M, Mingay LJ, Deng T, Sharps J (2002). A single amino acid mutation in the PA subunit of the influenza virus RNA polymerase inhibits endonucleolytic cleavage of capped RNAs.. J Virol.

[14] Fodor E, Mingay LJ, Crow M, Deng T, Brownlee GG (2003). A single amino acid mutation in the PA subunit of the influenza virus RNA polymerase promotes the generation of defective interfering RNAs.. J Virol.

[15] Kawaguchi A, Naito T, Nagata K (2005). Involvement of influenza virus PA subunit in assembly of functional RNA polymerase complexes.. J Virol.

[16] Mahy BWJ, Palese P, Kingsbury DW (1983). Mutants of influenza virus.. Genetics of influenza viruses.

[17] Huarte M, Falcon A, Nakaya Y, Ortin J, Garcia-Sastre A (2003). Threonine 157 of influenza virus PA polymerase subunit modulates RNA replication in infectious viruses.. J Virol.

[18] Hara K, Schmidt FI, Crow M, Brownlee GG (2006). Amino acid residues in the N-terminal region of the PA subunit of influenza A virus RNA polymerase play a critical role in protein stability, endonuclease activity, cap binding, and virion RNA promoter binding.. J Virol.

[19] Regan JF, Liang Y, Parslow TG (2006). Defective assembly of influenza A virus due to a mutation in the polymerase subunit PA.. J Virol.

[20] Guu TS, Dong L, Wittung-Stafshede P, Tao YJ (2008). Mapping the domain structure of the influenza A virus polymerase acidic protein (PA) and its interaction with the basic protein 1 (PB1) subunit.. Virology.

[21] Obayashi E, Yoshida H, Kawai F, Shibayama N, Kawaguchi A (2008). The structural basis for an essential subunit interaction in influenza virus RNA polymerase.. Nature.

[22] He X, Zhou J, Bartlam M, Zhang R, Ma J (2008). Crystal structure of the polymerase PA(C)-PB1(N) complex from an avian influenza H5N1 virus.. Nature.

[23] Dias A, Bouvier D, Crepin T, McCarthy AA, Hart DJ (2009). The cap-snatching endonuclease of influenza virus polymerase resides in the PA subunit.. Nature.

[24] Yuan P, Bartlam M, Lou Z, Chen S, Zhou J (2009). Crystal structure of an avian influenza polymerase PA(N) reveals an endonuclease active site.. Nature.

[25] Neumann G, Watanabe T, Ito H, Watanabe S, Goto H (1999). Generation of influenza A viruses entirely from cloned cDNAs.. Proc Natl Acad Sci U S A.

[26] Liang Y, Hong Y, Parslow TG (2005). cis-Acting packaging signals in the influenza virus PB1, PB2, and PA genomic RNA segments.. J Virol.

[27] Liang Y, Huang T, Ly H, Parslow TG, Liang Y (2008). Mutational analyses of packaging signals in influenza virus PA, PB1, and PB2 genomic RNA segments.. J Virol.

[28] Huarte M, Sanz-Ezquerro JJ, Roncal F, Ortin J, Nieto A (2001). PA subunit from influenza virus polymerase complex interacts with a cellular protein with homology to a family of transcriptional activators.. J Virol.

[29] Gabriel G, Dauber B, Wolff T, Planz O, Klenk HD (2005). The viral polymerase mediates adaptation of an avian influenza virus to a mammalian host.. Proc Natl Acad Sci U S A.

[30] Akkina RK, Chambers TM, Londo DR, Nayak DP (1987). Intracellular localization of the viral polymerase proteins in cells infected with influenza virus and cells expressing PB1 protein from cloned cDNA.. J Virol.

[31] Hemerka JN, Wang D, Weng Y, Lu W, Kaushik RS (2009). Detection and Characterization of Influenza a Virus Pa-Pb2 Interaction through a Bimolecular Flouresence Complementation Assay.. J Virol.

[32] Nieto A, de la Luna S, Barcena J, Portela A, Valcarcel J (1992). Nuclear transport of influenza virus polymerase PA protein.. Virus Res.

[33] Huet S, Avilov SV, Ferbitz L, Daigle N, Cusack S (2010). Nuclear import and assembly of influenza A virus RNA polymerase studied in live cells by fluorescence cross-correlation spectroscopy.. J Virol.

[34] Kumar N, Xin ZT, Liang Y, Ly H, Liang Y (2008). NF-kappaB signaling differentially regulates influenza virus RNA synthesis.. J Virol.

[35] Zurcher T, de la Luna S, Sanz-Ezquerro JJ, Nieto A, Ortin J (1996). Mutational analysis of the influenza virus A/Victoria/3/75 PA protein: studies of interaction with PB1 protein and identification of a dominant negative mutant.. J Gen Virol.

